# Bariatric Surgery in Adults with Obesity: the Impact on Performance, Metabolism, and Health Indices

**DOI:** 10.1007/s11695-020-05182-z

**Published:** 2021-01-17

**Authors:** Georges Jabbour, Ahmad Salman

**Affiliations:** Sport Science Program, College of Arts and Sciences, Qatar University, P.O. Box 2713, Doha, Qatar

**Keywords:** Obesity, Bariatric surgery, Cardiovascular, Metabolic, Aerobic, Cardiac autonomic function

## Abstract

This systematic review summarizes current evidence on the impact of bariatric surgery (BS) on physical performance, metabolic, and health indices in adults with obesity. This systematic review suggests that BS induced significant reductions in body weight, fat mass, and fat-free mass in individuals with obesity. Additionally, BS may improve many physical fitness and health indicators. Observed improvements manifest during a distinct period of time. To date, studies on BS and performance have been small in number, nonrandomized in design, and not controlled regarding gender distribution and/or post-surgery follow-up. Future studies should further investigate concerns associated with understanding of BS outcomes to improve these outcomes with potential benefits for quality of life, disability, mortality, morbidity, and overall BS success.

## Introduction

Severe obesity, defined as a body mass index (BMI) of at least 35 kg m^2^ [1], is strongly associated with several health complications [2–4] along with significant impairments in physical capacity and overall fitness parameters [5–8]. Bariatric surgery (BS) is emerging as an important option for those suffering from severe obesity when nonsurgical weight loss methods have been exhausted. In addition to the direct impact on weight loss, BS improves many health indicators during the post-operative period [9–13]. These changes were correlated with the quality of life and overall health parameters [13].

Changes attributed to BS at post-operative stages have focused mainly on body weight and composition changes, metabolic control, and energy adaptation [9, 10, 14–17] alongside some research that has investigated physical functioning and fitness capacity outcomes. These latter outcomes are known to be relevant in the obesity context especially since they are considered important mediators in developing risk factors for cardiovascular disease in this population [18–20].

In light of what was discussed above, this systematic review aimed to summarize recent findings on the effects of BS alone, without any exercise prescription or lifestyle modification, on the most relevant cardiorespiratory (e.g., oxygen uptake, heart rate), performance (e.g., muscular strength, distance covered), and health (e.g., autonomic nervous system modulation, metabolic parameters) outcomes in adults with obesity undergoing BS.

A good understanding of the effects of BS on cardiorespiratory, performance, and health outcomes is highly recommended for future intervention studies to improve these outcomes with potential benefits for quality of life, disability, mortality, morbidity, and overall BS success.

## Methods

### Eligibility Criteria

This systematic review was conducted in accordance with the Preferred Reporting Items for Systematic Reviews and Meta-Analyses (PRISMA) statement [21]. The population, intervention, comparator, outcomes, and study design (PICOS) approach was used to identify the inclusion criteria (Table 1). Only studies with a longitudinal design, of any duration, that have examined effects of BS on anthropometric characteristics and body composition (e.g., body weight, body fat, body mass index), physical performance (e.g., muscular strength, physical capacity), cardiorespiratory fitness and function (e.g., oxygen uptake, heart rate, heart rate variability), and energy expenditure and metabolism parameters (e.g., total energy expenditure, insulin resistance, lipid oxidation), in individuals with obesity undergoing any recognized surgical bariatric procedure, were eligible for inclusion. Studies were included in the current systematic review if they were in accordance with the following criteria: (1) published in peer-reviewed journals; (2) included adults and older of both genders; (3) compared BS outcomes at pre- and at post-surgery. Studies were excluded if they (1) assessed other types of interventions (in addition to the surgery), (2) reported only subjective measures, or (3) were not written in English. Moreover, review articles were not included in the current systematic review.

**Table 1:** PICOS criteria for the inclusion of studies

Parameters	Inclusion criteria
Population	Adults with severe obesity
Intervention	Bariatric surgery (purely gastric restrictive and gastric bypass with intestinal transposition)
Comparator	Pre-surgery versus post-surgery
Outcomes	Body composition, weight loss, physical capacity and performance, physical activity level, cardiorespiratory fitness, energy expenditure, metabolic parameters, substrate use, autonomic nervous system modulation
Study design	Retrospective, randomized control trial, and prospective studies

### Literature Search Strategy

Literature searches were conducted in four electronic databases, including PubMed, ISI Web of Knowledge, Web of Science, and SPORTDiscus. The following key terms (and synonyms searched for by the MeSH database) were included and combined using the operators “AND,” “OR,” and “NOT”: “anthropometric characteristics” or “body composition” or “physical performance” or “physical capacity” or “fitness” or “physical activity level” or “functional capacity” or “muscular performance” or “muscular strength” or “anaerobic capacity” or “aerobic capacity” or “cardiorespiratory function” or “cardiopulmonary function” or “energy expenditure” or “respiratory quotient” or “energy metabolism” or “cardiac autonomic control” or “heart rate variability” or “metabolic parameters” or and “bariatric surgery” or “obesity surgery” or “weight loss surgery” or “metabolic surgery” or “gastric bypass” or “gastric banding” or “sleeve gastrectomy” or “biliopancreatic diversion” or “duodenal switch.” The search was completed with a manual search of reference lists from key papers. Since the scope of this review is large in terms of outcome measures, a systematic review and not a meta-analysis was performed.

### Study Selection

The final screening was performed by the principal investigator (GJ) based on the relevance of the inclusion and exclusion criteria and the identified items for assessing the effects of BS on anthropometric characteristics and body composition (e.g., body weight, body fat, body mass index), physical performance (e.g., muscular strength, physical capacity), cardiorespiratory fitness and function (e.g., oxygen uptake, heart rate, heart rate variability), and energy expenditure and metabolism parameters (e.g., total energy expenditure, insulin resistance, lipid oxidation), in adults with obesity of both gender undergoing BS using PICOS criteria. If the citation showed any potential relevance, the abstract was screened. When abstracts indicated potential inclusion, full-text articles were reviewed.

## Results

### Study Selection and Description

**Fig. 1:**
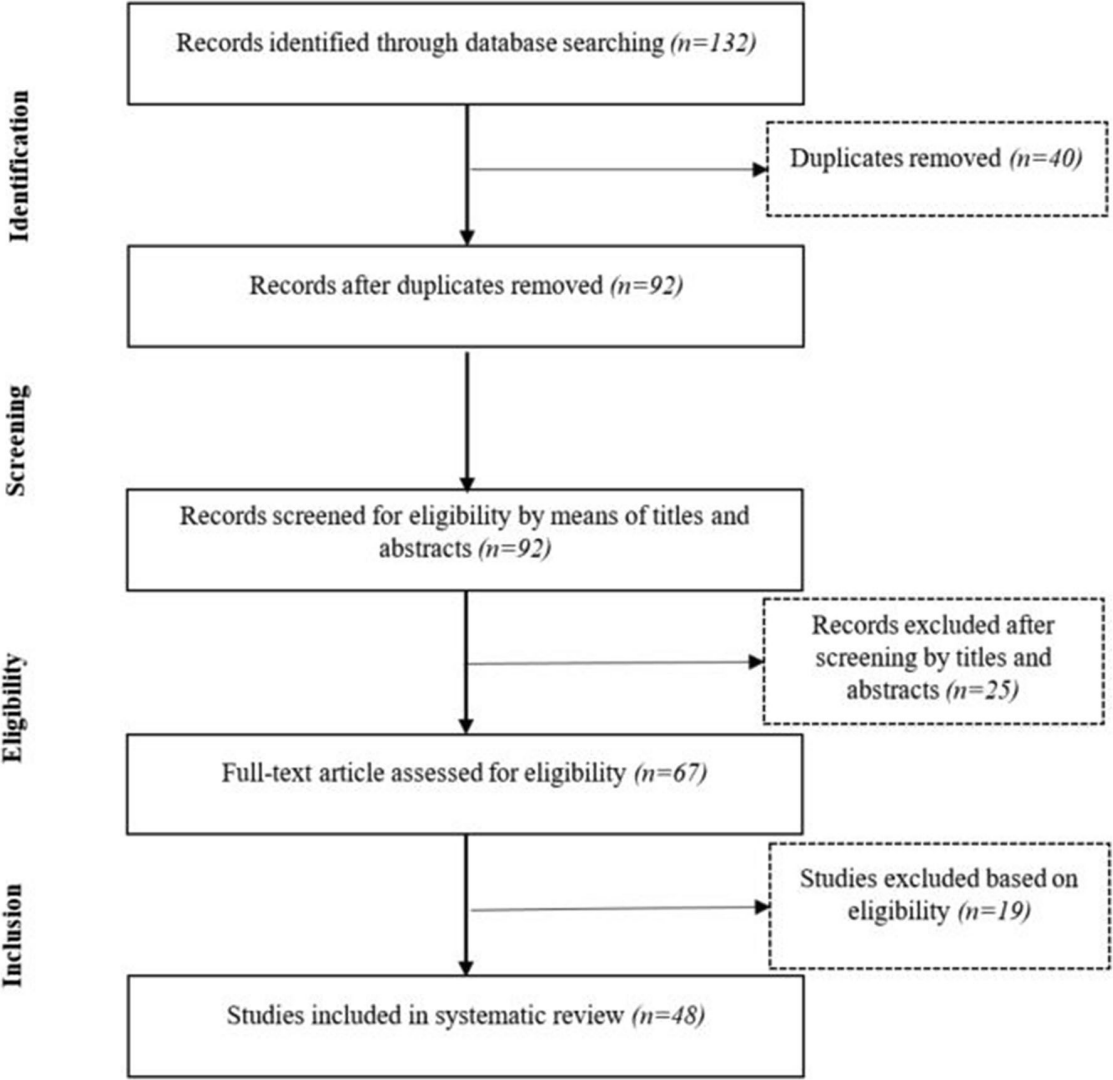
Flow diagram of included and excluded studies included in this systematic review using the recommendations in the Preferred Reporting Items for Systematic Reviews and Meta-Analyses (PRISMA) statement [21]

Our search initially identified 132 records (Fig. 1). After screening titles, abstracts, and full texts, 48 studies were included in our final analysis, and the characteristics of these long-term studies are shown in Table 2. The 48 studies reported on a total of 7105 patients; the mean age ranged from 18 to 60 years, and the mean follow-up interval ranged from 1 week to ≥ 24 months (Table 2). All studies had patient samples with a majority of female patients, except Wu et al. [65] who had two similarly sized gender groups (9 F and 9 M). The body mass index reported at baseline ranged from 37 to 55 kg/m^2^ (Table 2). Thirty-four studies used a gastric bypass (GB) procedure or a version of Roux-en-Y gastric bypass (RYGB) [16, 17, 22, 24–27, 30–32, 34, 36, 38–40, 42–45, 48–50, 52, 53, 55–61, 63, 64, 67], and seven studies reported laparoscopic adjustable gastric banding (LAGB) [22, 25, 33, 41, 43, 58, 67], of which five were combined with another BS method [22, 25, 33, 67]. Thirteen studies reported on laparoscopic sleeve gastrectomy (LSG) [28, 30, 36, 37, 47, 49, 52, 55, 56, 58, 59, 64], of which 8 were combined with another BS method [28, 30, 36, 49, 52, 55, 56, 58, 59, 64]. Three studies enrolled patients undergoing vertical-banded gastroplasty (VBG) [26, 35, 62], and Nault et al. [46] included patients who underwent BDP and biliopancreatic diversion.

**Table 2:** Baseline characteristics of studies included in the systematic review

Author (year)	Study design	Operation	Baseline BMI (SD)	Population		Evaluation period	Main outcomes
				Mean age, years (SD)	Gender		
Alam et al. [22]	Prospective cohort study	BPD (*N* = 5) LAGB (*N* = 6)	53.0 (7.3) BDP 44.2 (3.2) LGB	48 (8)	8 F and 3 M	Pre-surgery and 1, 6, and 12 months post-surgery	Body weight Autonomic nervous system modulation Metabolic parameters
Alba et al. [23]	Prospective cohort study	RYGB	44 (8)	45 (12)	37 F and 10 M	Pre-surgery and 6 and 12 months post-surgery	Body composition Physical capacity and performance Physical activity level
Benedetti et al. [14]	Prospective study	BPD	Not specified	36.1 (1.62)	9 F and 5 M	Pre-surgery and 30 months post-surgery	Body weight Body composition Energy expenditure Metabolic parameters
Bobbioni-Harsch et al. [24]	Prospective cohort study	RYGB	44.6 (1.1)	39.5 (2)	12 F	Pre-surgery and 3 and 12 months post-surgery	Body weight Body composition Metabolic parameters Autonomic nervous system modulation
Bond et al. [25]	Prospective cohort study	LAGB (*N* = 65%) RYGB (*N* = 35%)	50.1 (9.1)	46.2 (9.8)	17 F and 3 M	Pre-surgery and 6 months post-surgery	Physical activity level
Braga et al. [26]	Prospective cohort study	VBG (*N* = 5) RYGB (*N* = 14) Medical treatment (*N* = 20)	41.5 (5.0)	38.5 (10.6)	14 F and 6 M	Pre-surgery and 3 months post-surgery	Body weight Body composition Metabolic parameters Autonomic nervous system modulation
Browning et al. [27]	Prospective cohort study	GBS	42.9 (4.1)	21–55	9 F	Pre-surgery and 3 months post-surgery	Body weight Body composition Cardiorespiratory fitness
Campos et al. [9]	Prospective cohort study	Not specified	47.42 (5.72)	40 (7)	24 F	Pre-surgery and 6 months post-surgery	Body weight Body composition Physical capacity and performance Cardiorespiratory fitness Physical activity level
Carrasco et al. [16]	Prospective cohort study	RYGB	44.4 (4.8)	37.3 (11.1)	27 F and 4 M	Pre-surgery and 6 months post-surgery	Body weight Body composition Metabolic parameter Energy expenditure Substrate use Physical activity level
Colles et al. [17]	Prospective cohort	LAGB	44.3 (6.8)	45.2 (11.5)	103 F and 26 M	Pre-surgery and 4 and 12 months post-surgery	Body weight Body composition Physical activity level
Daniel et al. [28]	Prospective cohort study	LSG	44.04 (5.84)	47.0 (9.0)	14 F and 10 M	Pre-surgery and 5.9 (2.3) months and 15.5 (7.2) months post-surgery	Body weight Cardiorespiratory fitness
Das et al. [29]	Prospective cohort study	GBS	50.1 (9.3)	39.0 (9.6)	24 F and 6 M	Pre-surgery and 14 months post-surgery	Body weight Body composition Energy expenditure Physical activity level
Dereppe et al. [30]	Prospective cohort study	LSG (*N* = 18) RYGB (*N* = 24)	44 (4)	42 (13)	42 F	Pre-surgery and 12 months post-surgery	Body weight Body composition Metabolic parameters Cardiorespiratory fitness
De Souza et al. [31]	Prospective cohort study	RYGB	49.4 (5.4)	40.4 (8.4)	61 F and 4 M	Pre-surgery and 6 and 12 months post-surgery	Body weight Physical capacity and performance Cardiorespiratory fitness
De Souza et al. [32]	Prospective cohort study	RYGB	51.1 (9.2)	40.9 (9.2)	44 F and 7 M	Pre-surgery and 7 and 12 months post-surgery	Body weight Physical capacity and performance
Galtier et al. [33]	Prospective cohort study	LAGB	44.37 (7.0)	39.17 (10.4)	73 F	Pre-surgery and 13.37 (6.0) months post-surgery (group A [6–12 months, *n* = 39]; group B [12–18 months, *n* = 21]; group C [418 months, *n* = 13])	Body weight Body composition Metabolic parameters Energy expenditure Substrate use
Iannelli et al. [34]	Prospective cohort study	RYGB	44.6 (5.2)	39.9 (10)	115 F	Pre-surgery and 12 months post-surgery	Body weight Body composition Metabolic parameters Energy expenditure
Kanoupakis et al. [35]	Prospective cohort study	VBG	49 (8)	22–43	10 F and 6 M	Pre-surgery and 6 months post-surgery	Body weight Cardiorespiratory fitness
Kokkinos et al. [36]	Prospective cohort study	RYGB LAGB	48.4 (8.2)	44.2 (10.8)	450 F and 128 M	Pre-surgery and 12 months post-surgery	Body weight Body composition Autonomic nervous system modulation Cardiorespiratory fitness
Li et al. [37]	Prospective cohort study	RYGB (*N* = 14) LSG (*N* = 23)	47.9 (6.0) for the RYGB group 51.6 (7.5) for the SG group	38.0 (7.8) for the RYGB group 40.3 (9.9) for the SG group	Not specified	Pre-surgery and 3 and 6 months post-surgery	Body weight Body composition Metabolic parameters Cardiorespiratory fitness Autonomic nervous system modulation
Liu et al. [38]	Retrospective cohort study	LSG	37.2 (6.1)	35.3 (11.8)	52 F and 45 M	Pre-surgery and 6 months post-surgery	Body weight Body composition Energy expenditure Physical activity level
Lund et al. [39]	Prospective cohort study	GBS	44.6 (1.2)	41.2 (2)	11 F and 2 M	Pre-surgery and 6 months post-surgery	Body weight Body composition Metabolic parameters Cardiorespiratory fitness Physical activity level
Machado et al. [40]	Prospective cohort study	GBS	43 (1)	40 (2)	22 F and 9 M	Pre-surgery and 4 and 18 months post-surgery	Body weight Body composition Autonomic nervous system modulation
Maniscalco et al. [41]	Prospective cohort study	LAGB	43 (37 to 56)	37 (18 to 66)	42 F and 29 M	Pre-surgery and 6 months post-surgery	Body weight Body composition Autonomic nervous system modulation Physical capacity and performance
Maser et al. [42]	Prospective cohort study	RYGB	51 (11)	38 (11)	29 F and 3 M	Pre-surgery and 6 months post-surgery	Body weight Metabolic parameters Autonomic nervous system modulation
Maser et al. [43]	Prospective cohort study	RYGB	47.7 (7)	45 (9)	22 F and 4 M	Pre-surgery and 6 and 12 months post-surgery	Body weight Metabolic parameters Autonomic nervous system modulation
McCullough et al. [44]	Prospective cohort study	RYGB	50.4 (6.0)	46.0 (10.4)	82 F and 27 M	30-day period after discharge	Body weight Cardiorespiratory fitness
Mirahmadian et al. [45]	Prospective cohort study	RYGB (*N* = 21) LAGB (*N* = 5)	47.7 (7)	45 (9)	22 F and 4 M	Pre-surgery and 6 and 12 months post-surgery	Body weight Body composition Energy expenditure
Nault et al. [46]	Randomized controlled trials	BPD	52.3 (7.6) for BPD-DS and 54.3 (10.9) for C	37.7 (8.5) for BPD-DS and 44.7 (10.8) for C	6 F and 4 M for BPD-DS and 7 for C	Pre-surgery and 6 and 12 months post-surgery	Body weight Body composition Metabolic parameters Autonomic nervous system modulation
Neunhaeuserer et al. [47]	Prospective cohort study	LSG	45.2 (5.8)	48.23 (9.01)	26	Pre-surgery and 6 months post-surgery	Body weight Cardiorespiratory fitness Physical capacity and performance
Notarius et al. [48]	Randomized controlled trials	GBS	46.1 (6.4)	18–60	42 and 21 for C	Pre-surgery and 6 months post-surgery	Body weight Energy expenditure Cardiorespiratory fitness Physical capacity and performance
Otto et al. [49]	Prospective cohort study	RYGB (*N* = 16) LSG (*N* = 9)	47.40 (6.3)	36.8 (11.7) for F and 46.7 (9.0) for M	16 F and 9 M	Pre-surgery and repeated every 6 weeks for 4 months	Body weight Body composition Physical capacity and performance
Perugini et al. [50]	Prospective cohort study	RYGB	46 (6)	45 (9)	21 F and 7 M	Pre-surgery and 6 months post-surgery	Body weight Autonomic nervous system modulation Metabolic parameters
Ravelli et al. [51]	Prospective cohort study	RYGB	44.9 (2.5)	20 W	29.4 (5.1)	Pre-surgery and 6 and 12 months post-surgery	Body weight Body composition Energy expenditure
Remígio et al. [52]	Prospective cohort study	RYGB LSG	46.2 (4.9)	20–45	24	Pre-surgery and 4 months post-surgery	Body weight Metabolic parameters Cardiorespiratory fitness
Sans et al. [53]	Prospective cohort study	RYGB	43.3 (4.9)	40.6 (11.2)	103 F	Pre-surgery and 12 months post-surgery	Body weight Body composition Metabolic parameters Energy expenditure
Seres et al. [54]	Prospective cohort study	Not specified	51 (4)	38 (8)	20 F and 11 M	Pre-surgery and 12 months post-surgery	Body weight Cardiorespiratory fitness Physical capacity and performance
Schneider et al. [55]	Randomized controlled trials	RYGB LSG	43.9 (1.3)	40.3 (10.9) RYGB versus 41.2 (10.4) LSG	35 F and 7 M	Pre-surgery and 17 ± 5.6 months post-surgery	Body weight Body composition Energy expenditure Substrate use
Silva et al. [56]	Prospective cohort study	RYGB (*N* = 15) LSG (*N* = 2)	46 (2)	30 (1)	13 F and 4 M	Pre-surgery and 3 months post-surgery	Body weight Body composition Physical capacity and performance
Tamboli et al. [57]	Prospective cohort study	RYGB	46.3 (5.5)	43.8 (9.6)	25 F and 4 M	Pre-surgery and 6 and 12 months post-surgery	Body weight Body composition Energy expenditure Substrate use
Tam et al. [58]	Prospective cohort study	RYGB (*N* = 5) LSG (*N* = 9) LAGB (*N* = 7)	47.2 (1.5)	46 (2)	27 W	Pre-surgery and 8 weeks and 12 months post-surgery	Body weight Energy expenditure Physical activity level
Tettero et al. [59]	Retrospective cohort study	RYGB (*N* = 4359) LSG (*N* = 426)	44.9 (6.2)	43.1 (10.7)	3867 F and 918 M	Pre-surgery and 12 months post-surgery	Body weight Cardiorespiratory fitness Physical activity level
Tompkins et al. [60]	Prospective cohort study	LGB	45.5 (6.9)	44 (6.3)	28 F and 2 M	Pre-surgery and 6 and 12 months post-surgery	Body weight Body composition Physical capacity and performance
Valezi-Machado et al. [61]	Prospective cohort study	RYGB	41.8 (4.4)	35.9 (12.2)	31 F and 12 M	Pre-surgery and 12 months post-surgery	Body weight Cardiorespiratory fitness
Van Gemert et al. [62]	Prospective cohort study	VBG	48.1 (7.0)	28 (7)	7 W and 1 M	Pre-surgery and 3, 6, and 12 months post-surgery	Body weight Energy expenditure Substrate use
Vargas et al. [63]	Prospective cohort study	RYGB	50.45 (8.5)	38 (10)	61 W and 6 M	Pre-surgery and 3 months post-surgery	Body weight Physical capacity and performance
Wilms et al. [64]	Prospective cohort study	RYGB (*N* = 16) LSG (*N* = 2)	46.3 (6.8)	42.5 (10.6)	11 F and 7 M	Pre-surgery and 12 months post-surgery	Body weight Cardiorespiratory fitness Physical capacity and performance
Wu et al. [65]	Prospective cohort study	LSG	45.4 (6.8)	34	9 F and 9 M	Pre-surgery and 7, 30, 90, and 180 days post-surgery	Body weight Autonomic nervous system modulation Metabolic parameters
Zavorsky et al. [66]	Prospective cohort study	GBS	47.3 (6.2)	39 (8)	11 F and 4 M	Pre-surgery and 2 months post-surgery	Body weight Body composition Cardiorespiratory fitness

*BDP*, biliopancreatic diversion; *LAGB*, laparoscopic adjustable gastric banding; *RYGB*, Roux-en-Y gastric bypass; *VBG*, vertical-banded gastroplasty; *GBS*, gastric bypass surgery; *LSG*, laparoscopic sleeve gastrectomy; *F*, female; *M*, male; *C*, control group

Out of 48 studies, 43 were prospective cohorts [9, 14, 16, 17, 22–37, 39–45, 48–54, 56–58, 61–64, 66] and compared pre-operative to post-operative outcomes in adults undergoing BS. Mirahmadian et al. [45], Nault et al. [46], and Schneider et al. [55] were the only randomized control trials. While Mirahmadian et al. [45] and Nault et al. [46] compared patients who were receiving BS with a control group (without BS), Schneider et al. [55] examined whether there were differences between 2 surgical procedures, laparoscopic sleeve gastrectomy (LSG) and Roux-en-Y gastric bypass (RYGB), in terms of their effect on body composition and energy metabolism. The remaining two studies were retrospective cohorts that compared the main outcomes pre- and post-surgery [45, 46].

### Post-operative Body Composition Changes and Weight Loss

Due to the research context, all of the studies include post-operative body composition and weight loss as their primary outcome. Body composition changes and weight loss were generally reported as FM (%, kg), FFM (%, kg), BW (kg), BMI (kg/m^2^), AC (cm), waist circumference (cm), hip circumference (cm), and W/H ratio. All studies reported a significant improvement in post-operative body composition and weight loss (Tables 3 and 4). These improvements were detected at different post-operative follow-up periods [9, 14, 16, 24, 26, 27, 33, 34, 37, 39, 45, 55, 66]. Some studies examined FFM changes over different post-operative periods and reported significant decreases in FFM (kg) values after a short post-operative follow-up (2 months) for up to 2 years.

**Table 3:** Post-operative body composition, weight loss, physical activity level, performance, cardiorespiratory fitness, energy expenditure, metabolic parameters, substrate use, and autonomic nervous system modulation

Author (year)	Methods	Results	Post-surgery evaluation period (month)												
			1	2	3	4	5	6	7	12	14	16	17	18	≥ 24
Alam et al. [22]	RR and QT time series	↓BW (kg)	•					•		•					
		↓BMI (kg/m^2^)													
		↓HR (bpm) (at 6th)													
		↑QTVI (1st and 12th)													
		↓SampEn (QT) (at 1st)													
		↓DFAα (NN) (1st) ↓DFAα (QT) (1st)													
		↓RR (6th)													
		↓RMSSD (ms) (at 6th)													
		↓SDNN (ms) (at 6th)													
		↓HbA1c (%) (at 12th) ↓Rest systolic blood pressure (mmHg) (at 6th and 12th)													
		↓ Rest diastolic blood pressure (mmHg) (at 12th)													
Alba et al. [23]	Gait speed and time to rise from a chair five times + 400-m walk test + Handgrip strength + International Physical Activity Questionnaire (IPAQ)	↓FFM (kg) ↑Gait speed ↑Time for five chair stands ↓Absolute grip strength (at 6th and 12th) ↑Relative strength (at 6th and 12th) ↔ Self-reported physical activity						•		•					
Benedetti et al. [14]	Respiration chamber	↓FM (kg) ↓FFM (kg) ↓REE ((kcal/24 h)) ↑Fasting npRQ ↓Fasting glucose (mmol/dl) ↓Fasting insulin (mU/ml) ↓Fasting FFA (mM)													•
Bobbioni-Harsch et al. [24]	Body impedance analyzer + A 120-min euglycemic, hyperinsulinemic clamp + Plasma levels of glucose and free fatty acids (FFA) were enzymatically determined + Heart rate variability (HRV): electrocardiograph continuously recorded for a 24-h period	↓BMI (kg/m^2^) ↓FM (kg) ↓FFM (kg) ↑Glucose uptake (mg/kg LBM/min) ↔ FFA (mEq) ↓Plasma insulin (ng/ml) ↑SDNN (ms) ↑RMS (ms) ↑% pNN 50			•					•					
Bond et al. [25]	RT3 accelerometers + Paffenbarger Physical Activity Questionnaire (PPAQ)	55% comply with the recommendation (subjective assessment) versus 5% comply with the recommendation (objective assessment)						•							
Braga et al. [26]	Digital scale and a tape measure + HOMA-IR and glucose were quantified by the glucose oxidase colorimetric method + Endothelial reactivity and HRV analysis were performed by peripheral arterial tonometry (PAT)	↓FM (kg) ↓FFM (kg) ↓AC (cm) ↔ HOMA-IR (%) ↓Fasting glucose (mg/dl) ↓Fasting insulin (IU/l) ↔ LnRHI ↔ AI ↔ AI@75 ↓HR (bpm) ↑HRV-time domain ↓HRV-frequency domain (↑LF/HF) ↔ Systolic blood pressure (mmHg) ↔ Diastolic blood pressure (mmHg)			•										
Browning et al. [27]	Treadmill with gas-exchange analysis + Stanford 7-day Physical Activity Recall (PAR)	↓BMI (kg/m^2^) ↓BW (kg) ↓FM (kg) ↓FFM (kg) ↓Submaximal HR (bpm) ↔ VO_2_ (l/min/kg) ↑Time to exhaustion (min) ↔ HRmax (bpm) ↔ Absolute VO_2peak_ (l/min) ↑VO_2peak_ (ml/kg/min) ↔ RERmax ↑VO_2peak_/pulse (ml/beat/kg) ↔ Post-exercise La (mmol/l)			•										
Campos et al. [9]	Magnetic bioimpedance device + Lung function: computerized ultrasound spirometer with a flow sensor + Respiratory muscle strength: analog manometer + Functional capacity: incremental shuttle walk test (ISWT) + Baecke questionnaire	↓BMI (kg/m^2^) ↓BW (kg) ↓WC (cm) ↔ W/H ↓FM (kg) ↓FFM (kg) ↑SVC (l) ↑FVC (l) ↑FEV1 (l) ↑MIP (cmH_2_O) ↔ MEP (cmH_2_O) ↑Distance (m) ↔ PA level						•							
Carrasco et al. [16]	Digital scale and a scale-mounted stadiometer + isotopic dilution with deuterium oxide (total body water) + Open-circuit indirect calorimetry using a ventilated chamber system + Simple survey to assess PA + HOMA-IR	↓BMI (kg/m^2^) ↓BW (kg) ↓WC (cm) ↓FM (kg) ↓FFM (kg) ↓W/HR ↓Fasting glucose (mg/dl) ↓Fasting insulin (μU/ml) ↓HOMA-IR ↓Total cholesterol (mg/dl) ↓LDL cholesterol (mg/dl) ↑HDL cholesterol (mg/dl) ↓Triglycerides (mg/dl) ↓Systolic blood pressure (mmHg) ↓Diastolic blood pressure (mmHg) ↓REE ↑Fasting lipid oxidation (%) ↑PA level						•							
Colles et al. [17]	Medical Outcomes Trust Short Form-36 (SF-36)	↓BW (kg) ↓BMI (kg/m^2^) ↑SF-36 PCS score (at 12th)				•								•	
Daniel et al. [28]	Treadmill with gas-exchange analysis	↓BMI (kg/m^2^) ↑METs max ↑Exercise time (s) ↑VO_2peak_ (ml/min/kg) ↓VO_2peak_ (ml/min) Tau (τ) altered at 6th and improved at 16th ↔ RERmax ↑HR/VO_2_ slope (at 6th) ↓HR rest (bpm) (at 6th) ↓Rest systolic blood pressure (mmHg) (at 6th) ↓Rest diastolic blood pressure (mmHg) (at 6th)						•						•	
Das et al. [29]	15-day doubly labeled water; indirect Calorimetry + Minnesota Leisure Time Physical Activity (LTPA) questionnaire (structured interview)	↓BMI (kg/m^2^) ↓BW (kg) ↓FM (kg) ↓FM (%) ↓TEE (MJ/day) ↓REE (MJ/day) ↔ Physical activity level (TEE/REE) ↔ Reported activity (min/day)									•				
Dereppe et al. [30]	Graded cycle ergometer with gas-exchange analysis	↓BW (kg) ↓BMI (kg/m^2^) ↓ Rest systolic blood pressure (mmHg) ↓ Rest diastolic blood pressure ↓Glucose (mg/dl) ↑HDL cholesterol (mg/dl) ↓LDL cholesterol (mg/dl) ↓Triglycerides (mg/dl) ↓VO_2peak_ (ml/min) ↑VO_2peak_ (ml/min/kg) ↓W (W) ↑RERmax								•					
De Souza et al. [31]	Treadmill with gas-exchange analysis	↓BW (kg) ↓BMI (kg/m^2^) ↑Distance covered (m) ↑Exercise duration (min) ↑VO_2max_ (ml/kg/min)						•		•					
De Souza et al. [32]	6-min walk test	↓BW (kg) ↓BMI (kg/m^2^) ↑Distance covered (m) ↓Perceived exhaustion ↓HR ↓Respiratory frequency							•						
Galtier et al. [33]	Indirect calorimetry with gas-exchange analysis + HOMA-IR + Bioimpedance analysis	↓BW (kg) ↓BMI (kg/m^2^) ↓WC (cm) ↓ Rest systolic blood pressure (mmHg) ↓ Rest diastolic blood pressure (mmHg) ↓Fat-free mass (kg) ↓Fat mass (%) ↔ Fasting blood glucose (mmol/l) ↓120-min OGTT blood glucose (mmol/l) ↓Fasting plasma insulin (mIU/l) ↓Peak-OGTT plasma insulin (mIU/l) ↓Total cholesterol (mmol/l) ↓Triglycerides (mmol/l) ↑HDL cholesterol (mmol/l) ↔ LDL cholesterol (mmol/l) ↓HOMA-IR ↓REE/FFM ↑REE/BW ↔ Lipid oxidation						• A		• A, B				• B	
Iannelli et al. [34]	Bioelectrical impedance analysis + Wall-mounted stadiometer and a digital electronic scale + Indirect calorimetry with gas-exchange analysis	↓BW (kg) ↓BMI (kg/m^2^) ↓WC (cm) ↓FFM (kg) ↓FM (%) ↓REE (kcal/24 h) ↓Glucose levels (mmol/l) ↓HOMA-IR ↓HbA1c (%) ↑HDL cholesterol (mmol/l) ↓LDL cholesterol (mmol/l) ↓Triglycerides (mmol/l)								•					
Kanoupakis et al. [35]	Treadmill with gas-exchange analysis + M-mode, 2-dimensional, and Doppler echocardiography	↔ Rest HR (bpm) ↔ Rest systolic blood pressure (mmHg) Anaerobic threshold ↔ HR (bpm) ↔ Systolic blood pressure (mmHg) ↓VO_2_ (ml/min) ↑VO_2_ (ml/kg/min) ↔ O_2_ pulse (ml/beat) Maximal exercise ↔ HR (beats/min) ↔ Systolic blood pressure (mmHg) ↑Time (s) ↓VO_2_ (ml/min) ↑VO_2_ (ml/kg/min) ↔ O_2_ pulse (ml/beat) ↑Ventilation (l/min) ↑VCO_2_ production (ml/min) ↑METs ↓IVS (mm) ↓PW (mm) ↑E/A ↓IVRT (ms)						•							
Kokkinos et al. [36]	Heart rate variability (HRV) (frequency domain) + Echocardiography	↓BMI (kg/m^2^) (at 3th and 6th) ↓Waist (cm) (at 3th and 6th) ↓Hip (cm) (at 3th and 6th) ↑LF (ms^2^) (for SG) ↑HF (ms^2^) (for SG and GB) ↔ LF/HF ratio ↑Total power (ms^2^) (for SG and GB) ↓Epicardial fat (mm) (at 6th) (for SG and GB) ↓LV Tei index (at 6th month) (for SG and GB) ↓LA diameter (mm) (at 6th) (for SG and GB) ↑EF (%) (at 6th) (for SG and GB) ↑LV mass index (g) (at 6th) (for SG and GB)			•			•							
Li et al. [37]	Glucose oxidase method + High-performance liquid chromatography + Automatic analyzer + Electronic scale and fixed wall stadiometer + Segmental bioelectrical impedance analysis + Gas-exchange analysis	↓BW (kg) ↓BMI (kg/m^2^) ↓WC (cm) ↓FFM (kg) ↓FM (kg) ↓Rest systolic blood pressure (mmHg) ↔ Rest diastolic blood pressure (mmHg) ↓Total cholesterol (mmol/l) ↓Triglycerides (mmol/l) ↑HDL cholesterol (mmol/l) ↓LDL cholesterol (mmol/l) ↓Blood glucose levels ↓HbA1c (%) ↓RQ ↓REE (kcal) ↓REE/BW ↔ REE/FFM						•							
Liu et al. [38]	Bioelectrical impedance analysis + Dual-energy x-ray absorptiometry + Treadmill with gas-exchange analysis + Accelerometer	↓BW (kg) ↓BMI (kg/m^2^) ↓WC (cm) ↓Fat mass (kg) ↓REE (kcal/day) ↓REE/FFM ↔ Physical activity level						•							
Lund et al. [39]	Stationary ergometer bike with gas-exchange analysis + Physical function was assessed by the SF-36 questionnaire + CAMB questionnaire	↓BW (kg) ↓BMI (kg/m^2^) ↓FFM (kg) ↓FM (%) ↓Fasting insulin (pmol/l) ↓Fasting glucose (mmol/l) ↓HbA_1_c (mmol/mol) ↓Fasting total cholesterol (mmol/l) ↓Systolic blood pressure (mmHg) ↔ Diastolic blood pressure (mmHg) ↓VO_2_ (ml/min) ↑VO_2_ (ml/kg/min) ↔ VO_2_ (ml/kgFFM/min) ↔ Exercise (h/week) ↔ Physical activity level		•		•								•	
Machado et al. [40]	Electronic anthropometric scale + Heart rate variability (HRV) (time domain)	↓BMI (kg/m^2^) ↓WC (cm) ↑NN (ms) ↑SDNN (ms) ↑PNN50 (%) ↑RMSSD (ms)						•							
Maniscalco et al. [41]	Lung volumes and flow rates were determined using automated equipment + 6-min walk test + Oximeter	↑FVC (% pred) ↑FEV1 (% pred) ↔ FVC/FEV1 ↑TLC (% pred) ↑FRC (% pred) ↑RV (% pred) ↑6-mWT distance (m) ↑HR after 6-mWT (b/min) ↑Baseline SaO_2_ (%) ↔ SaO_2_ after 6-mWT (%) ↓Dyspnea score after 6-mWT								•					
Maser et al. [42]	Measures of HRV (e.g., power spectral analysis, RR variation during deep breathing) + HOMA-IR	↓BW (kg) ↓HOMA-IR ↓LF ↓HF ↓LF/HF ↑Respiration frequency area						•							
Maser et al. [43]	RR (interval between R waves of electrocardiographic QRS complexes) + Stadiometer + Finger stick blood glucose readings + Hemoglobin A1c was measured by high-performance ion-exchange liquid chromatography	↓BMI (kg/m^2^) ↓Fingerstick glucose (mg/dl) ↓HbA1c (%) ↓Systolic blood pressure (mmHg) ↔ Diastolic blood pressure (mmHg) ↑MCR ↓E/I ratio ↑Valsalva ratio						•		•					
McCullough et al. [44]	Bruce treadmill protocols with gas-exchange analysis	↓BMI (kg/m^2^) ↔ Systolic blood pressure (mmHg) ↔ Diastolic blood pressure (mmHg) ↑Exercise duration (min) ↑Maximal HR (beats/min) ↔ Perceived exertion (Borg, 6–20) ↓VO_2 peak_ (l/min) ↑VO_2 peak_ (ml/kg/min) ↑V-AT (ml/kg/min) ↔ VE/VCO_2_ slope	•												
Mirahmadian et al. [45]	Indirect calorimeter with gas-exchange analysis	↓Body weight (kg) ↓BMI (kg/m^2^) ↓Fat-free mass (kg and %) ↓Fat mass (kg and %) ↓REE (kcal/day) ↑REE/FM (kcal/kg)			•										
Nault et al. [46]	Heart rate variability (HRV) (time domain and frequency domain) + Echocardiogram + Biochemical analysis + HOMA-IR	↓Body weight (kg) ↓BMI (kg/m^2^) ↓Total cholesterol (mmol/l) ↓Triglycerides (mmol/l) ↑HDL cholesterol (mmol/l) ↓LDL cholesterol (mmol/l) ↓Glucose (mmol/l) ↓Insulin (pmol/l) ↓HOMA-IR ↓HR (beats/min) ↑SDNN (24 h) ↑rMSSD (24 h) ↑pNN50 (24 h) ↑Ln LF (ms^2^) (24 h) ↑Ln HF (ms^2^) (24 h) ↓LF/HF (24 h)						•		•					
Neunhaeuserer et al. [47]	Treadmill with gas-exchange analysis + One-repetition maximum (1-RM)	↑Exercise time (s) ↓VO_2peak_ (l/min) ↑VO_2peak_ (ml/kg/min) ↑VO_2_/HRmax (ml/bpm) ↓OUES (ml/logl) ↔ RERmax ↑Leg extension (kg) ↔ Handgrip right (kg) ↔ Handgrip left (kg)						•							
Notarius et al. [48]	Treadmill with gas-exchange analysis	↓TEE ↑Exercise capacity ↓VO_2peak_ (ml/kg/min)						•							
Otto et al. [49]	Bioelectrical impedance + Handgrip strength	↓BMI (kg/m^2^) ↓Fat-free mass (kg) ↓Fat mass (%) ↓Fat mass (kg) ↔ Handgrip strength (kg) dominant hand ↔ Handgrip strength (kg) no dominant hand		•		•									
Perugini et al. [50]	Heart rate variability (HRV) + HOMA-IR	↓BW (kg) ↓BMI (kg/m^2^) HRV (improved) ↓HOMA-IR						•							
Ravelli et al. [51]	Doubly labeled water + Triaxial accelerometer	↓BW (kg) ↓FM (%) ↓TEE						•		•					
Remígio et al. [52]	Treadmill with gas-exchange analysis	↓BW (kg) ↓BMI (kg/m^2^) ↓Systolic blood pressure (mmHg) ↓Diastolic blood pressure (mmHg) ↓Resting HR (bpm) ↓Total cholesterol (mmol/l) ↓LDL cholesterol (mmol/l) ↓Triglycerides (mmol/l) ↔ Glucose (mg/dl) ↔ VO_2peak_ (l/min) ↑VO_2peak_ (ml/min/kg) ↓50%VO_2_ RP (s)				•									
Sans et al. [53]	Homeostasis model assessment of insulin resistance (HOMA-IR) + Bioelectrical impedance analysis (BIA) + Gas-exchange analysis	↓BW (kg) ↓BMI (kg/m^2^) ↓WC (cm) ↓HC (cm) ↔ W/H ↓ Brachial circumference (cm) ↓Triceps skinfold thickness (cm) ↓Glucose level (mmol/l) ↓Insulin level (mmol/l) ↓HOMA-IR ↓HbA1c (%) ↑HDL cholesterol (mmol/l) ↓LDL cholesterol (mmol/l) ↓Triglyceride (mmol/l) ↓REE (kcal/day) ↑REE/BW ↓REE/FFM								•					
Seres et al. [54]	Treadmill with gas-exchange analysis	↑Exercise duration (min) ↑HRmax (bpm) ↑RERmax ↔ VO_2peak_ (l/min) ↑VO_2peak_ (ml/kg/min) ↔ VO_2peak_/FFM (ml/kg/min) ↔ VO_2peak_/pulse (ml/beat) ↔ Minute ventilation (l/min)								•					
Schneider et al. [55]	Dual-energy X-ray absorptiometry + Indirect calorimetry	↓BW (kg) ↓BMI (kg/m^2^) ↓FFM (kg) ↓FM (%) ↓REE ↑REE/BW ↓Fat oxidation ↔ CHO oxidation											•		
Silva et al. [56]	Handgrip dynamometer + Venous occlusion plethysmography + 6-min walk test	↓BW (kg) ↓BMI (kg/m^2^) ↓HR (bpm) ↔ Systolic blood pressure (mmHg) ↔ Diastolic blood pressure (mmHg) ↓FVR (units) ↔ 30% handgrip force (Kgf) ↑6-mWT distance (m) ↓Apnea-hypopnea index			•										
Tamboli et al. [57]	Digital scale + A whole-room indirect calorimeter	↓BW (kg) ↓BMI (kg/m^2^) ↓WC (cm) ↓W/H (at 6th month) ↓TEE (kcal/day) (at 6th month) ↓Total RQ (at 6th month) ↓Sleep RQ (at 12th month) ↓CHO oxidation (g/kg/day) (at 12th month) ↑Fat oxidation (g/kg/day) (at 12th month)						•		•					
Tam et al. [58]	Metabolic chamber indirect calorimetry	↓24hrEE ↓SleepEE ↓REE ↓Spontaneous physical activity		•						•					
Tettero et al. [59]	Baecke questionnaire + Astrand test	↓BW (kg) ↑VO_2max_ (ml/min/KgFFM) ↑ Leisure physical activity ↑ Sport activity								•					
Tompkins et al. [60]	Physical ability using SF-36 + 6-mWT + Borg RPE scale	↓BW (kg) ↓BMI (kg/m^2^) ↑6-mWT distance (m) ↑Physical functioning ↓Rating of perceived exertion during 6-mWT			•			•							
Valezi-Machado et al. [61]	Treadmill with gas-exchange analysis + Transthoracic echocardiogram	↑Distance covered (m) ↑METs ↑VO_2peak_ (ml/kg/min) ↑EF ↓Septum								•					
Van Gemert et al. [62]	Doubly labeled water method + Respiration chamber	↓TEE ↓Sleep MR ↔ Physical activity index =[TEE/SMR] ↓CHO oxidation			•					•					
Vargas et al. [63]	6-min walking test + Functional Independence Measure (FIM) + Timed Up-and-Go	↓HR (bpm) ↓Respiratory rate (pm) ↓Systolic arterial pressure (mmHg) ↓Diastolic arterial pressure (mmHg) ↓Borg scale ↑FIM score			•										
Wilms et al. [64]	Bicycle ergospirometry	↔ Peak workload (W) ↑Peak workload/BW (W kg^−1^) ↔ Test duration (s) ↔ HRmax (bpm) ↔ RERmax ↔ VO_2peak_ (l/min) ↔ VO_2peak_ (ml/kg/min) ↔ VO_2peak_/pulse (ml/beat) ↔ Ventilatory equivalent (VE/VO_2_)													•
Wu et al. [65]	Heart rate variability (HRV) + Insulin resistance + HbA1c	RMSSD improved LF/HF ratio improved ↑Total power ↓HOMA-IR ↓HbA1c	•		•	•									
Zavorsky et al. [66]	Bioelectrical impedance device + Ergocycle with gas-exchange analysis	↓BW (kg) ↓BMI (kg/m^2^) ↓WC (cm) ↓HC (cm) ↓W/H ↓FFM (kg) ↓FM (kg) ↓FM (%) Rest ↔ VO_2_ (ml/kg/min) ↓VO_2_ (l/min) ↓VE (l/min) BTPS ↔ Breathing frequency (breaths/min) ↓Tidal volume (l/breath) ↓VErest/MVV ↓RER ↓HR (bpm) At peak exercise ↑VO_2_ (ml/kg/min) ↔ VO_2_ (l/min) ↔ VE (l/min) BTPS ↔ Breathing frequency (breaths/min) ↑Tidal volume (l/breath) ↔ VEpeak/MVV ↔ RER ↔ HR (beats/min) ↔ Total time of the VO_2peak_ test			•										

*A*, peak late of diastolic filling wave velocity; *AC*, abdominal circumference; *AI*, augmentation index; *AI@75*, AI index standardized for a heart rate of 75 bpm; *BMI*, body mass index; *BTPS*, body temperature and pressure saturated; *BW*, body weight; *E*, peak early of diastolic filling wave velocity; *E/A*, velocity ratio; *E/I*, expiration/inspiration; *EF*, ejection fraction; *ERV*, expiratory reserve volume; *FEV1*, forced expiratory volume in first second; *FFA*, free fatty acids; *FFM*, fat-free mass; *FM*, fat mass; *FRC*, functional residual capacity; *FVC*, forced vital capacity; *FVR*, forearm vascular resistance; *HbA1c*, glycated hemoglobin; *HF*, high frequency; *HOMA-IR*, homeostatic model assessment for insulin resistance; *HR*, heart rate; *IC*, inspiratory capacity; *IRV*, inspiratory reserve volume; *IVRT*, isovolumic relaxation time; *IVS*, interventricular septum; *La*, lactate; *LA*, left atrium; *LF*, low frequency; *LF/HF*, low to high frequency ratio; *LnRHI*, reactive hyperemia index; *LV*, Left ventricle; *MCR*, mean circular resultant; *MEP*, maximal expiratory pressure; *MET*, metabolic equivalent of task; *MIP*, maximal inspiratory pressure; *MVV*, maximum voluntary ventilation; *npRQ*, non-protein respiratory quotient; *O*_*2*_*-p*, oxygen pulse; *OGTT*, oral glucose tolerance test; *OUES*, oxygen uptake efficiency slope = (the slope of linear regression of VO_2_ (L/m) versus log VE (L/m)); *pNN 50* (ms), percentage of adjacent NN intervals that differ from each other by more than 50 ms; *PW*, posterior wall thickness; *QTVI*, temporal behavior of the QT variability index; *REE*, resting energy expenditure; *RMSSD*, root mean square of the successive differences; *RQ*, respiratory quotient; *SampEn*, measures of the complexity; *SaO*_*2*_, oxygen saturation; *SDNN*, standard deviation of NN intervals; *SMR*, sleeping metabolic ratio; *SVC*, slow vital capacity; *TEE*, total energy expenditure; *TLC*, total lung capacity; *V-AT*, ventilatory-derived anaerobic threshold; *VE/VCO*_*2*_, the minute ventilation/carbon dioxide production; *VO*_*2*_, oxygen uptake; *W*, watt; *W/H*, waist-to-hip ratio; *WC*, waist circumference, *50%VO*_*2*_ *RP*, Post-exercise Oxygen Uptake Recovery Kinetics; ↑ denotes a significant increase; ↓ denotes a significant decrease; ↔, no change

**Table 4:** Main analyzed parameters of performance and health indices and type of bariatric surgery

	Main analyzed parameters							
	Body weight	Body mass index	Resting energy expenditure	Total energy expenditure	Heart rate variability	Aerobic capacity	Physical capacity	Plasma insulin
Type of bariatric surgery								
BDP [14, 22, 46]	↓ [14, 22, 46]	↓ [14, 22, 46]	↓ [14]	-	↑ [14, 46]	-	-	↓ [14, 46]
LAGB [17, 22, 25, 33, 36, 41, 45, 58]	↓ [17, 22, 33, 45]	↓ [17, 22, 33, 36, 45]	↓ [17, 33, 45, 58]	-	↑ [36]	↑ [41]	↑ [41]	↓ [17, 33]
RYGB [16, 23–26, 30–32, 34, 36, 37, 42–45, 49–53, 55–57, 59, 61, 63, 64]	↓ [16, 23, 24, 26, 30–32, 34, 37, 42, 45, 50–53, 55–57, 59]	↓ [16, 24, 30–32, 34, 36, 37, 43, 45, 49, 50, 52, 53, 55–57]	↓ [34, 37, 45, 53, 55]	↓ [51, 57]	↑ [24, 26, 36, 42, 50]	↑ [30, 31, 44, 52, 59, 61]	↑ [23, 31, 32, 44, 49, 56, 59, 61, 63, 64]	↓ [16, 24, 53]
VBG [26, 35, 62]	↓ [26]	↓ [26]	-	↓ [62]	↑ [26]	↑ [35]	↑ [35, 62]	-
GBS [27, 29, 39, 40, 48, 60, 66]	↓ [27, 29, 39, 60, 66]	↓ [27, 29, 39, 40, 60, 66]	↓ [29, 66]	↓ [29, 48]	↑ [40]	↑ [27, 39, 66]	↑ [48, 60, 66]	↓ [39]
LSG [28, 30, 37, 38, 47, 49, 52, 55, 56, 58, 59, 64, 65]	↓ [28, 30, 37, 38, 52, 55, 56, 59]	↓ [30, 37, 38, 49, 52, 55, 56]	↓ [37, 38, 55, 58]	-	↑ [65]	↑ [28, 30, 47, 52, 59]	↑ [28, 47, 56, 59]	-

*BDP*, biliopancreatic diversion; *LAGB*, laparoscopic adjustable gastric banding; *RYGB*, Roux-en-Y gastric bypass; *VBG*, vertical-banded gastroplasty; *GBS*, gastric bypass surgery; *LSG*, laparoscopic sleeve gastrectomy; ↑ denotes a significant increase; ↓ denotes a significant decrease; -, not reported

### Post-operative Physical Activity Level and Performance

Twenty-four of 48 studies examined the impact of BS on many performance components (Tables 3 and 4) and/or on the post-surgery physical activity levels (Table 3) and used different assessment methods (objective and subjective) to compare outcomes with pre-surgery points. A majority of studies reported the impact of BS on exercise and functional capacity by evaluating various indices, such as gait speed and the time to rise from a chair five times [23]; the distance covered in meters [9, 31, 32, 41, 44]; exercise duration [28, 31, 35, 47, 54, 56, 61, 64, 66]; perceived exhaustion [32, 63]; and the Functional Independence Measure [63]. These studies reported a favorable impact of BS on these outcomes [9, 23, 28, 31, 32, 35, 41, 44, 47, 54, 56, 61, 63]. In contrast, only Wilms et al. [64] did not find any favorable effect of BS on the distance covered ≥ 24 months post-surgery. Muscular performance has been evaluated by reporting absolute and relative grip strength [23, 47, 49, 56], peak power, developed in Watts or relative to body weight [64], or leg extension performance [47]. Some results demonstrated that BS had a beneficial effect on grip strength [23] while other studies found no beneficial effect on grip strength, [47, 49, 56] and that it had a beneficial effect on leg extension performance [47] and on peak power relative to body weight [64].

Nine out of 48 of the selected studies assessed post-surgery physical activity levels and compared them to the pre-surgery period (Tables 3 and 4). Four of the studies used the validated Physical Activity Questionnaire [9, 23, 25, 39] to evaluate subjective physical activity levels and did not report any changes in the post-surgery period compared to baseline. One study that utilized self-developed surveys to assess physical activity [16] showed an increase in the physical activity (PA) level at the 6th month post-surgery evaluation. Bond et al. [25] compared subjective evaluations using the Paffenbarger Physical Activity Questionnaire (PPAQ) and objective measurements using a triaxial accelerometer. They reported that 55% of responders meet the international guideline recommendations when subjectively assessed versus 5% who meet these recommendations when objectively assessed. For Liu et al. [38], the PA level reported via accelerometer did not improve 6 months after BS. Das et al. [29], Tam et al. [58], and Van Germet et al. [62] used a metabolic chamber for indirect calorimetry during the post-surgery period and found no significant changes [29, 62] and even decreases [58] in the PA index among patients.

### Post-operative Cardiorespiratory Fitness and Energy Expenditure

Details of the effects of BS on different cardiorespiratory fitness and energy indices expenditure are summarized in Tables 3 and 4. Eleven studies evaluated the effects of BS on cardiorespiratory capacity (oxygen consumption, oxygen uptake efficiency, heart rate max, ventilatory equivalent, lung capacity, and breathing frequency) using a treadmill [27, 28, 31, 35, 44, 47, 48] or an ergometer [30, 39, 64, 66].

Of these 14 studies, 11 reported a significant increase in VO_2peak_ relative to body weight [27, 28, 30, 31, 35, 39, 44, 47, 52, 54, 61], and 5 reported no change [27, 30, 52, 54, 64] or a decrease [28, 30, 35, 39, 44, 47, 66] in absolute VO_2peak_ (7 studies). Only two studies reported a decrease [48] or no change [64] in VO_2peak_ relative to body weight. Other parameters, such as oxygen uptake efficiency, decreased [47], while ventilatory response [66], and ventilatory volume and efficiency [54, 64] improved post-surgery.

The change in total energy expenditure (TEE) between the pre-operative period and follow-up was reported in four studies [29, 48, 51, 60]. Compared with the pre-operative value, the TEE decreased at 6, 12, and 14 months post-operatively. Ten studies [16, 29, 33, 34, 37, 38, 45, 53, 55, 58] reported a reduction in resting energy expenditure (REE) post-surgery. REE/BW was reported in four studies [33, 37, 53, 55], and REE/FFM was reported in five studies [33, 37, 38, 45, 53].

There were significant increases [33, 53, 55] and decreases [37] in REE/BW after BS. REE/FFM decreased [33, 38, 53], increased [45], or did not change [37] after BS.

Five studies [16, 33, 55, 57, 62] reported changes in substrate oxidation during the pre-operative and follow-up period. Compared with the pre-operative value, CHO oxidation decreased at the 3rd [57, 62] and 12th month [57, 62] post-surgery or had not changed at the 14th month post-surgery [55]. In terms of fat oxidation, Carrasco et al. [16] reported a significant increase in fasting lipid oxidation at 6 months post-surgery and a decrease [55] at 17 months post-surgery, and Tamboli et al. reported a decrease at 12 months post-surgery. In contrast, no changes were reported by Galtier et al. [33] at the 6th, 12th, and 18th months post-surgery [33].

### Post-operative Metabolic Parameters, Substrate Use, and Autonomic Nervous System Modulation

At ≥ 24 months post-surgery, Benedetti et al. [14] reported significant improvements in metabolic parameters manifested by decreases in fasting glucose, insulin, and FFA levels. For Bobbioni-Harsch et al. [24], plasma glucose and FFA remained unchanged post-surgery. However, plasma insulin decreased at both 3 months and 12 months. Glucose uptake increased at 3 months and 12 months post-surgery. Braga et al. [26] reported no changes in homeostatic model assessment for insulin resistance (HOMA-IR) (%) but a decrease in fasting glucose and insulin at 3 months post-surgery (Table 3). Lipid profiles (total cholesterol, LDL cholesterol, HDL cholesterol, and triglycerides) improved significantly at the 4th [52], 6th [16, 33], and 12th [30, 34, 37, 53] months post-surgery as did glucose and HbA1c levels [30, 34, 37, 43, 46, 53] and insulin resistance [33, 34, 46, 53] at the 12th and 6th months [42, 46, 50] post-surgery. Lund et al. [39] reported significant decreases in fasting insulin and glucose levels, as well as in HbA1c and fasting total cholesterol at the 2nd and 4th months post-surgery. Wu et al. [65] reported significant decreases in HOMA-IR and HbA1c at the 1st, 3rd, and 4th months post-surgery.

Alam et al. [22] reported an improvement in the temporal behavior of the QT variability index (QTVI) at the 1st and 12th months following BS. Three other indices (SampEn QT, DFAα (NN), and DFAα (QT)) also improved within 1 month following surgery, and a further four (RR, HR, RMSSD, and SDNN) showed an improvement at 6 months post-surgery. Bobbioni-Harsch et al. [24] reported an improvement in SDNN as well as RMS and % pNN 50 at all follow-up periods. An improvement in both the frequency and time domain has been reported by Braga et al. [26] at the 3rd month and by Nault et al. [46] at the 6th and 12th months post-surgery (Table 3). Kokkinos et al. [36] compared the SG versus GB surgery methods and reported an improvement in frequency domain variables regardless of the groups at 3 and 12 months post-surgery. The HRV-time domain [40] and HRV-frequency [42] domain indices improved at the 6th month [40] post-surgery, and both improved at 6 and 12 months for Nault et al. [46] and at the 1st, 3rd, and 4th months post-surgery for Wu et al. [65]

Other forms of improvement have been reported for heart structure using echocardiography. Two studies reported decreases in IVS, PW, and IVRT and increases in E/A at 6 months post-surgery [35] as well as decreases in epicardial fat, LV Tei index, and LA diameter and increases in EF (%) and LV mass index at 6 months post-surgery for both the SG and GB surgery groups. For endothelial reactivity, no changes were reported for LnRHI, AI, or AI@75 at the 3rd month post-surgery (Table 3) [26].

## Discussion

This systematic literature review indicates that undergoing bariatric surgery may procure several health benefits and improve some fitness and performance indicators regardless of the procedure. These improvements may be achieved after short- and/or mid-term post-operative periods. Despite these promising results, more consideration of candidate profiles prior to BS in addition to a longer follow-up with multiple visits is highly recommended to gain a fuller understanding of the influence of BS on the selected outcomes.

### Post-operative Body Composition Changes and Weight Loss

Despite heterogeneity in participant age, baseline BMI, the surgical procedure used, and the technique used to assess body composition, studies revealed significant reductions in body weight and fat mass and a decrease in fat-free mass in individuals with obesity who underwent BS.

The significant body weight loss reported by studies on BS was essentially attributed to reducing energy intake and decreased absorption of nutrients [16, 24]. However, it is important to mention that weight loss is also influenced by variations in the surgical technique, such as the size of the gastric pouch, the alimentary limb length, and the gastrojejunostomy diameter. In fact, the variation in the operative technique affects energy intake among patients leading to the inter-individual variability of weight loss after surgery. On the other hand, many authors suggested that the metabolic adaptation that accompanies weight loss, in addition to variations in plasma levels of mediators derived from adipose tissue, such as leptin, was related mostly to loss of fat mass rather than to a decrease in fat-free mass [16, 68, 69].

Although caloric restriction seems to be the dominant mechanism in body weight reduction and weight loss maintenance, Gemert et al. [62] suggested that a decrease in CHO intake resulted in lower insulin levels, which increased lipolysis and decreased CHO and protein oxidation, procures a beneficial effect on weight loss success.

To conclude, a multitude of factors may be involved in explaining body composition changes and weight loss among BS patients. Considering that an appropriate and permanent reduction in energy intake is essential for long-term weight management in patients who have undergone BS, examining other potential explanations for the variability in weight loss between patients will help potentiate short- and long-term weight loss post-bariatric surgery.

### Post-operative Physical Activity Levels and Performance

The effect of BS on post-surgery physical activity levels has been evaluated either subjectively using self-reported questionnaires [9, 23, 25], structured interviews [29], or simple surveys performed by the participants [16], or objectively using an accelerometer [25, 38]. There is a great variation in how exercise is measured and the minimal threshold to define a physically active patient. Of these subjective evaluations, the results reported no changes in physical activity levels during the post-surgery period [9, 23, 29, 39]. For Campos et al. [9], despite the reported improvements in body composition, cardio-respiratory performance, and functional capacity 6 months post-surgery, participants were still sedentary. These results might be related to a lack of consistency in performing physical exercise, as was experienced prior to surgery. Only one study by Carrasco et al. [16] reported an increase in physical activity and a decrease in sedentary behavior. This increase was related to weight loss. When using an accelerometer, Bond et al. [25] reported a near fivefold decrease in MVPA among participants compared to using a self-reported evaluation, and only one participant met the physical activity recommendations. Due to the lack of data on baseline variables regarding a “voluntary” change in physical activity level after bariatric surgery, how to determine post-surgery physical activity practices in bariatric surgery patients is still unknown.

Despite heterogeneity in participant age, baseline profile, surgical procedure used, and test performed, studies reported a positive impact of BS on many performance indicators among the patients. These improvements were related to muscular strength and physical function. For Alba et al. [22], relative muscle strength and physical performance improved between 6 and 12 months post-operatively despite declines in lean mass and absolute muscle strength. Moreover, Alba et al. [22] reported a significant improvement in physical performance, attributed to the person’s ability to perform activities of daily living, as reported recently by Campos et al. [9] among women with morbid obesity 6 months post-operatively after performing the incremental shuttle walk test (ISWT). De Souza et al. [32], Maniscalco et al. [41], Silva et al. [56], Tompkins et al. [60], and Vargas et al. [63] reported an increase in distance when performing a 6-min walking test (6-mWT) with a concomitant decrease in the rating of perceived exertion [32, 60, 63], and body mass and BMI decreases were the strongest predictors of that improvement [41, 56, 63].

A lower muscle strength was associated with the loss of lean body mass, which accompanied the reduction in fat mass, particularly in the first months after surgery [70, 71]. Future studies evaluating both muscle mass and function, as well as fiber-type composition, will help better address this issue.

### Post-operative Cardiorespiratory Fitness and Energy Expenditure

Individuals with severe obesity suffer from impaired cardiorespiratory fitness [72–74] that manifests primarily with a decreased VO_2peak_. In response to BS, studies reported significant increases in VO_2peak_ relative to body weight fitness [27, 28, 30, 31, 35, 39, 44, 47, 52, 54, 61, 66] and in lung function [9, 41, 66], suggesting improved aerobic fitness. However, absolute values were either unchanged [27, 52, 54, 64, 66] or decreased [28, 30, 35, 39, 44, 47]. In the absence of any scheduled physical conditioning or exercise intervention, the improvement in VO_2peak_ relative to weight as well as the decreases in absolute VO_2peak_ post-surgery is mainly attributed to weight loss and body composition changes. However, it is important to note that a significant proportion of weight loss following bariatric surgery comes from muscle mass, especially in the initial post-surgical period [71, 74], and oxidative muscle metabolism [70].

Therefore, it is unclear whether the increased aerobic capacity post-surgery reflects a fundamental improvement at the muscular and cardiorespiratory structure levels or is simply due to a lower energy requirement associated with exercise and reduced strain on the cardiopulmonary system during exercise. More recently, Daniel et al. [28] explored short- and long-term post-surgical effects on aerobic fitness parameters (absolute VO_2peak_, OUES, and the time constant Tau (τ) in VO_2_ kinetics) in a homogenous population after LSG. For these authors, the restoration of overall aerobic capacity could be achieved in the long-term post-surgery, allowing an improvement in overall aerobic performance. The latter will also depend on other physiological, environmental, and behavioral characteristics. Consequently, future studies should use multiple time points to give a better understanding as well as have an extended follow-up period, and they should consider other predictors of aerobic performance (e.g., stroke volume, aerobic enzyme, muscle fiber types) that are known to significantly affect these parameters.

Regarding REE, a meta-analysis by Astrup et al. [75] reported that post-operative weight loss is associated with a reduction in REE. For Carrasco et al. [16], the reduction in weight was associated with a significant decrease in the REE/FFM ratio, and greater decreases were shown for those with higher REEs at baseline. In this context, many studies supported that a greater energy expenditure at the pre-surgical stage might be compensatory for energy intake increases when restricted nutritional intake is applied in the post-operative state; this compensation would disappear, leading to a greater reduction in REE in patients with obesity [16, 29]. Another factor that would explain REE decreases in the post-surgical phase is fat mass loss. Carrasco et al. [16] observed a positive correlation between REE changes and the reduction in body fat at the 6-month follow-up. It seems that REE adaptation may be influenced by the reduction in adipose tissue and variations in plasma levels of mediators derived from this tissue in addition to other factors such favorable changes in eating habits, physical activity, and nutritional behavior [76–78], and on the absence of metabolic factors that predispose individuals to regain weight [77, 79].

In terms of REE, TEE decreased significantly post-surgery among patients with obesity [29, 51, 57]. For Tamboli et al. [57], the decrease in TEE did not appear to follow the same pattern as the REE change after BS. A decline in TEE was observed until 6 months post-surgery, while no significant difference was reported at 12 months post-operatively. This decrease was proportional to the weight change within 6 months after surgery, and no further change in TEE occurred with ongoing weight loss. One possible explanation for this is the change in the PA level and diet-induced thermogenesis.

### Post-operative Metabolic Parameters, Substrate Use, and Autonomic Nervous System Modulation

Studies have reported immediate improvements in metabolic parameters during the post-surgery period, mainly in the lipid profile (e.g., remission concerning the levels of total cholesterol, LDL cholesterol, and triglycerides) and glycemic control (e.g., improvement in the levels of fasting insulin and the HOMA index, normalization of fasting glycaemia levels, and increases in glucose uptake) [14, 16, 22, 24, 26, 30, 33, 34, 37, 39, 43, 46, 50, 52, 53, 65] and in substrate oxidation (e.g., increases in lipid oxidation and decreases in carbohydrate oxidation) [55, 57, 62]. It has been postulated that these improvements were mainly attributed to body composition changes, mainly to fat mass [16, 80], to visceral fat loss [33, 34], and to changes in intestinal peptides (GLP-1, PYY3–36, etc.) [81, 82], regardless of the surgical procedure.

Obesity alters heart rate variability (HRV) that will manifest as a decrease in HRV due to decreased adrenoreceptor responsiveness, withdrawal of parasympathetic (vagal) tone, and/or increased sympathetic activity [83, 84]. Weight loss improves parasympathetic cardiac modulation, observed as an increase in HRV [85]. Many studies have reported a significant association between weight loss and HRV improvement. For Alam et al. [22], several indices showed a prompt and persistent improvement with progressive weight loss, mainly for the QTVI, which improved as early as 1 month following surgery, and this change was further improved at the 12-month follow-up. Similarly, Maser et al. [42] showed that an average 28% reduction in BMI was accompanied by very significant improvements in all measures of HRV. Other factors, such as hormonal and metabolic factors, have been assessed to elucidate whether one or more of these factors are associated with modifications of cardiac autonomic balance post-surgery. For example, Bobbioni-Harsch et al. [24] showed that in addition to body weight loss, energy intake explained 20% of the variations in the time domain profile 3 months post-surgery. Kokkinos et al. [36] found that PHF and TP were both increased, indicating amelioration of cardiac autonomic function overall and the reversal of vagal impairment. Machado et al. [40] reported an overall HRV increase 6 months post-surgery, and this increase was more evident in men. Moreover, cardiac parasympathetic activity also increased but only in younger patients. Finally, HRV improvement was associated with lipid profile improvement at the 6th and 12th months post-surgery [46] and with insulin resistance decreases [65].

## Limitations

It is important to note that the evidence presented in this review comes from different BS’s procedures, e.g., metabolic versus restrictive, which compares different parameters difficult to interpret. While metabolic surgery, mainly gastric bypass and biliopancreatic diversion, is used to treat metabolic diseases, especially type 2 diabetes, the restrictive surgery is considered weight loss surgery. Considering that surgical technique is beyond the scope of this systematic review, however, most of our selected studies have been performed with patients who underwent a gastric bypass (GB) procedure, which may help sort out some interpretation. Moreover, studies were heterogeneous, and full descriptions of inclusion criteria and the adjustment by other covariates such as participant characteristics and duration of follow-up were not always reported. Finally, it is still important to mention that the lack of randomized control trial studies is really significant, and most of the studies recruited are very small in sample size in parallel to a short follow-up time which makes the results less appealing.

## Conclusion

This review summarizes the benefits of BS alone for several performance and health indicators in adults with obesity. A key conclusion is that BS has a positive impact on body composition, physical functioning, metabolic parameters, and autonomic nervous system modulation and, to some extent, on energy expenditure, physical activity level, muscular strength, and peak oxygen consumption. As an effective approach to reducing body weight when nonsurgical methods are exhausted, the improvements procured by BS have been achieved both in a shorter period (less than 1 month) and with more extended period (more than 1 year); however, some studies reported that some of these benefits might disappear later on. Therefore, further studies are needed to determine the appropriate recommendations that still imprecise until today, focusing on managing post-surgery outcomes mainly by considering lifestyle modification that is likely to be of significant benefit.

## Abbreviations

A: Peak late of diastolic filling wave velocity
AC: Abdominal circumference
AI: Augmentation index
AI@75: AI index standardized for a heart rate of 75 bpm
BMI: Body mass index
BS: Bariatric surgery
BTPS: Body temperature and pressure saturated
BW: Body weight
E: Peak early of diastolic filling wave velocity
E/A: Velocity ratio
E/I: Expiration/inspiration
EF: Ejection fraction
ERV: Expiratory reserve volume
FEV1: Forced expiratory volume in first second
FFA: Free fatty acids
FFM: Fat-free mass
FM: Fat mass
FRC: Functional residual capacity
FVC: Forced vital capacity
FVR: Forearm vascular resistance
HbA1c: Glycated hemoglobin
HF: High frequency
HOMA-IR: Homeostatic model assessment for insulin resistance
HR: Heart rate
IC: Inspiratory capacity
IRV: Inspiratory reserve volume
IVRT: Isovolumic relaxation time
IVS: Interventricular septum
La: Lactate
LA: Left atrium
LF: Low frequency
LF/HF: Low to high frequency ratio
LnRHI: Reactive hyperemia index
LV: Left ventricle
MCR: Mean circular resultant
MEP: Maximal expiratory pressure
MET: Metabolic equivalent of task
MIP: Maximal inspiratory pressure
MVV: Maximum voluntary ventilation
npRQ: Nonprotein respiratory quotient
O_2_-p: Oxygen pulse
OGTT: Oral glucose tolerance test
OUES: Oxygen uptake efficiency slope = (the slope of linear regression of VO_2_ (L/m) versus log VE (L/m))
pNN 50 (ms): Percentage of adjacent NN intervals that differ from each other by more than 50 ms
PW: Posterior wall thickness
QTVI: Temporal behavior of the QT variability index
REE: Resting energy expenditure
RMSSD: Root mean square of the successive differences
RQ: Respiratory quotient
SampEn: Measures of the complexity
SaO_2_: Oxygen saturation
SDNN: Standard deviation of NN intervals
SMR: Sleeping metabolic ratio
SVC: Slow vital capacity
TEE: Total energy expenditure
TLC: Total lung capacity
V-AT: Ventilatory-derived anaerobic threshold
VE/VCO_2_: The minute ventilation/carbon dioxide production
VO_2_: Oxygen uptake
W: Watt
W/H: Waist-to-hip ratio
WC: Waist circumference
50%VO_2_ RP: Post-exercise Oxygen Uptake Recovery Kinetics
